# Development, Reproduction, and Life Table Parameters of the Foxglove Aphid, *Aulacorthum solani* Kaltenbach (Hemiptera: Aphididae), on Soybean at Constant Temperatures

**DOI:** 10.3390/insects11050296

**Published:** 2020-05-11

**Authors:** Bo Yoon Seo, Eun Young Kim, Jeong Joon Ahn, Yonggyun Kim, Sungtaeg Kang, Jin Kyo Jung

**Affiliations:** 1 Crop Protection Division, National Academy of Agricultural Science, Rural Development Administration, Wanju-Gun, Jeollabuk-do 55365, Korea; seoby@korea.kr; 2 Crop Cultivation and Environment Research Division, National Institute of Crop Science, Rural Development Administration, Suwon-si, Gyeonggi-do 16429, Korea; key4082@korea.kr; 3 Research Institute of Climate Change and Agriculture, National Institute of Horticultural & Herbal Science, Rural Development Administration, Jeju-si, Jeju-do 63240, Korea; j2ahn33@korea.kr; 4 Department of Plant Medicals, Andong National University, Andong-si, Gyeongsangbuk-do 36729, Korea; hosanna@andong.ac.kr; 5 College of Biotechnology & Bioengineering, Dankook University, Cheonan-si, Chungcheongnam-do 31116, Korea; kangst@dankook.ac.kr

**Keywords:** *Glycine max*, *Aulacorthum solani*, temperature, development, reproduction, life table

## Abstract

We investigated several characteristics of the development and reproduction of the aphid *Aulacorthum solani* raised on soybean (*Glycine max*) at 10 constant temperatures between 2.5 and 30 °C, and described the relationship between temperature and several critical biological characteristics using mathematical models. We found that *A. solani* could survive and reproduce on soybean at temperatures ranging from 5 to 27.5 °C. High fecundity was observed at temperatures from 12.5 to 20 °C. The lower developmental threshold and thermal constant for this species’ nymphal stages were estimated to be 5.02 °C and 131.2 degree-days, respectively, using a linear model. The upper developmental threshold was estimated to be 33.9 °C using the Lactin-2 model. The optimum temperature for nymphal development was estimated to be 26.9 °C. The maximum total fecundity was estimated as ca. 76.9 nymphs per adult at 18.1 °C. The daily fecundity sharply increased at earlier adult ages, and slowly decreased thereafter until final parthenogenesis occurred, over a range of temperatures from 12.5 to 25 °C. The maximum daily fecundity was estimated to be ca. 6.1 nymphs per adult per day for a 5.2 day old of adult at 21.3 °C using an age- and temperature-dependent model of adult fecundity. In terms of life table statistics, the intrinsic rates of increase and the finite rate of increase were both highest at 25 °C, while the net reproductive rate was highest at 20 °C.

## 1. Introduction

The foxglove aphid, *Aulacorthum solani* (Hemiptera: Aphididae), is a highly polyphagous insect. The number of host plants on which it can feed is as high as 540 identified species (or 688, including unidentified ones) from 33 orders and 82 families throughout the world; in Korea, it has been recorded to feed on 26 (identified) and up to a maximum of 30 plant species (including unidentified ones) from 10 orders and 13 families [1]. This aphid is a major pest of soybean, *Glycine max* L. (Fabaceae), in Korea and Japan. It mainly feeds on the backsides of soybean leaves, and causes reduced yields of soybean through virus transmission and directly sucking out leaf fluids, as well as severe leaf yellowing [2,3,4]. This aphid occurs on plants from the seedling stage onward during the early summer season in Korean soybean fields, and its density in fields usually reaches maximum values during the fall, in the last half-season of the soybean’s reproductive stage. In the results of two sequential scouting studies, maximum densities in September in Korea were recorded as ca. 4300 aphids per 20 hills [2] or ca. 7.4 aphids per leaf [5].

The aphid can develop through both holocyclic and anholocyclic life cycles. The insect overwinters as eggs in the holocyclic cycle, or as nymphs or adults in the anholocyclic cycle on various host plants [6,7,8,9]. *A. solani* is a species that is better adapted to low temperatures than insects in other orders, like many other aphid species [10]. Temperature is one of the most important abiotic factors impacting developmental and reproductive processes in insects because they are poikilotherms, and it affects their survival, population dynamics, and distributions [11]. Many studies, therefore, have focused on the relationship between temperature and development in insect species [12,13,14].

A few studies have described experiments examining the effects of temperature on the development and reproduction of *A. solani* at multiple temperatures (ranging from low to high ones), derived general relationships between temperature and its development, and constructed life tables for the species when reared on lettuce (*Lactuca sativa* L.) (Asterales: Asteraceae) [15,16,17,18,19] and pansy (*Viola* × *wittrockiana)* (Malpighiales: Violaceae) [20]. However, only one previous study investigated some biological properties of the development and reproduction of *A. solani* on soybean at multiple temperatures between 10 and 35 °C, and did not perform statistical analyses on their data [2]. Kingsolver et al. [21] pointed out that mean thermal performance can differ in fluctuating and constant thermal environments. They showed that *Manduca sexta* L. larvae reared in diurnally fluctuating temperatures had significantly higher optimal temperatures and maximal growth rates than larvae reared in constant temperatures. Considering constant temperature has the advantage of allowing direct effects of temperature on the development, longevity, and fecundity of insects to be measured.

Therefore, in this study, we used a wider range of temperatures, from 2.5 to 30 °C, in order to (1) investigate the developmental characteristics of *A. solani* on soybean at different temperatures; (2) describe the generalized relationships between temperature and biological traits, development, and fecundity in this species using mathematical models commonly used in arthropod species; and (3) construct life tables for this species to understand the biological characteristics when reared on soybean. We used the traditional age-specific life table and the age–stage, two-sex life table developed by Chi and Liu [22] and Chi [23]. Although *A. solani* populations are parthenogenetic, we applied the age–stage, two-sex life table to precisely present the stage differentiation among individuals and then used the life tables to project the population growth at different temperatures. The results of this study can support future studies of this aphid’s population phenology, adaptation to climate change, and performance on different soybean cultivars.

## 2. Materials and Methods

### 2.1. Insect Colony

Nymphs and adults of *A. solani* were collected from soybean fields in Suwon, Korea, in 2014, and non-parasitized clones were selected through individual rearing in the laboratory. The clones were combined and reared successively on seedlings of the soybean cultivar Taegwangkong in an acrylic plastic cage (27.5 cm width × 30 cm depth × 40 cm height) in a plant growth chamber. The environmental conditions of the chamber were maintained at 20 ± 3 °C temperature, 15:9 h (light:dark) photoperiod, and 60% ± 15% relative humidity. During successive rearing processes, a small proportion of alate form was observed depending on density.

Several apterous adults in the rearing colony were transferred to soybean leaves in Petri dishes (50 mm diameter × 15 mm height) held in the same rearing conditions, and then nymphs newly born on the next day (after ca. 24 h) were used for rearing experiments at different temperatures. For adult experiments at 30 °C only, late-instar nymphs from the rearing colony were transferred to soybean leaves in other Petri dishes, and the apterous adults that emerged the next day were used. Experiments were conducted during the period from 2015 to 2017.

### 2.2. Investigation of Nymph Development and Adult Reproduction at Constant Temperatures

Ten constant temperatures (2.5, 5.0, 7.5, 10.0, 12.5, 15.0, 20.0, 25.0, 27.5, and 30 °C) were examined, with aphids in each temperature treatment held at the same photoperiod (15:9 h = light:dark). A low-temperature incubator (Dasol Ltd., Suwon, Korea) was used to rear aphids at temperatures from 2.5 to 15 °C, while a four-chamber incubator (Dasol Ltd., Suwon, Korea) was used for rearing at all other temperatures. Each neonate nymph was reared on a single leaf of soybean (cultivar Taegwangkong) within a polystyrene Petri dish (50 mm diameter × 15 mm height). The petiole of the leaf was wrapped in moistened cotton wool, and small drops of distilled water were applied to the cotton wool every day to maintain the leaf’s freshness. Each leaf was exchanged with a new one at 4–7 day intervals. Thirty replicated nymphs were used for each temperature. The occurrence of developmental milestones such as molting, death, and reproduction was recorded daily until death. Neonate nymphs produced from adults were removed immediately after being counted. For the adult experiment at 30 °C, thirty apterous adults were treated without examination of their nymphal stages.

Mean values and standard deviations (SD) for the duration of the total nymphal period, each instar period, adult longevity, pre-reproductive period, reproductive period, total fecundity, and daily fecundity during the reproductive period were calculated. One-way analyses of variance and comparisons of significant differences among different temperatures via Tukey’s HSD test (*α* = 0.05) were conducted using the PROC GLM function in the program of SAS Ver. 9.1 [24]. Stage-specific mortality during the nymphal period was calculated as the percentage of the initial number of nymphs present in a stage that later died during that stage (i.e., the number that died divided by the initial number). Reproductive rate was calculated as the percentage of the initial number of adults that produced progeny.

### 2.3. Models and Estimation of Parameters

The characteristics investigated at each temperature were analyzed using several mathematical models that produced development curves similar in shape to the observed data, and containing parameters and derivatives with biological meanings. The parameters of all two-dimensional models were estimated with TableCurve 2D Ver. 5.01 (Systat Software Incorporated, San Jose, USA), while those of the three-dimensional model (Equation (8)) were estimated with TableCurve 3D Ver. 4.0 (Systat Software Incorporated, San Jose, USA).

The relationship between temperature and the development rate of *A. solani* nymphs was modeled using both a linear model and the non-linear Lactin-2 empirical model. Nymphal development rates, *r*(*T*_c_), at a given temperature (in °C), *T_c_*, were calculated as the reciprocals of the individual nymphs’ developmental periods (days) during the stage; these values were then averaged for each temperature. In the linear model [25] (Equation (1)), only data from the temperature range between 7.5 and 25 °C, over which the relationship between temperature (*x*-axis) and development rate (*y*-axis) was linear, were used to estimate the model’s parameters. In the linear model, the lower development threshold (°C) (LDT) and degree-days (DD) above the LDT required for the completion of development (i.e., the thermal constant) of the nymphal stages were calculated as −*b*/*a* and 1/*a*, respectively, based on the parameters in the linear equation as presented below.

(1) $$r\left(T_{C}\right)=aT_{c}+b$$

In the nonlinear Lactin-2 model (Equation (2)) [26], data from all temperatures at which development was successfully completed were used to estimate model parameters, where *T_L_* is the upper developmental threshold (UDT), *ΔT* is the temperature range over which fast physiological breakdown occurs near UDT, and *λ* is a parameter that is derived to make the development curve intersect the abscissa at a suboptimal temperature; the formula for this model is shown below.

(2) $$r\left(T_{c}\right)=e^{\left(\rho T_{c}\right)}-e^{\left(\rho T_{L}-\frac{T_{L}-T_{c}}{\Delta T}\right)}+\lambda$$

In this model, the optimal temperature (*T*_opt_), at which the maximum development rate was predicted to occur, was calculated as *T_L_* − *ΔT*. The LDT was obtained as the approximate *T*_c_ value when *r*(*T*_c_) = 0; this calculation was performed with the parameters estimated in Equation (2) using Microsoft Mathematics Version 4.0.

A two-parameter Weibull equation (Equation (3)) was used to model inter-individual differences in the completion of development [14,27]. In this model, *F*(*x*) is the cumulative proportion of nymphal development completed at a normalized time, *x*, corresponding to development time divided by the mean developmental period, and *η* and *β* are fitted parameters, as follows.

(3) $$F\left(x\right)=1-e^{-{\left(x/\eta \right)}^{\beta }}$$

Aging rate, *r*(*A*_c_), at a particular temperature, *T* (in °C), during the adult stage was calculated as the mean value of the reciprocals of all individual longevities. The temperature-dependent aging rate was fitted with an Eyring model (Equation (4)) previously used for *Rhopalosiphum padi* (Hemiptera: Aphididae) [28,29,30], where *a* and *b* are fitted parameters as shown below.

(4) $$r\left(A_{c}\right)=aTe^{-b/T}$$

The relationship between total fecundity per female and temperature was estimated using Equation (5), proposed by Briere et al. [31].

(5) $$f\left(T\right)=aT\left(T-T_{L}\right){\left(T_{M}-T\right)}^{1/m}$$

where *f*(*T*) is the number of total nymphs laid by one female at each temperature, *T_L_* and *T_M_* are the lower and higher temperatures for oviposition, and *a* and *m* are empirical constants of the equation.

The relationship between cumulative fecundity ratio and the normalized adult age (=adult age/mean adult longevity) was fitted with the two-parameter Weibull model used for describing the completion of nymphal development (Equation (3)) above.

The relationship between cumulative survival ratio, *s*(*x*), and the normalized adult age, *x*, was described with a model (Equation (6)) previously used for *Carposina sasaki* (Lepidoptera: Crambidae) [32], in which *γ* is the normalized age at 50% cumulative survival rate and *δ* is a fitted parameter, as follows.

(6) $$s\left(x\right)=\frac{1}{1+e^{\left(\gamma -x\right)/\delta }}$$

The relationship between daily fecundity, *y*, and adult age, *x*, at the same temperature was described with an adjusted nonlinear model (Equation (7)) previously used for *Myzus persicae* (Hemiptera: Aphididae) [33], in which *p*_1_ is the total fecundity, *p*_2_ is the mean pre-reproductive period, and *p*_3_ is a speed constant, as follows.

(7) $$y=p_{3}{}^{2}p_{1}\left(x-p_{2}\right)e^{-p_{3}\left(x-p_{2}\right)}$$

The relationship between temperature, *T*, adult age, *x*, and daily fecundity, *F*(*x*)*_T_*, was described with a model (Equation (8)) previously used for a mite species, *Hypoaspis miles* (Mesostigmata: Laelapidae) [34], in which *ϵ*, *θ*, *κ*, *ρ*, *ν*, and *ψ* are fitted parameters, as shown below.

(8) $$F{\left(x\right)}_{T}=e^{\left(\epsilon +\theta T^{2}+\kappa T^{3}\right)}xe^{-\left(e^{\left(\rho +\nu T^{2}+\psi T^{3}\right)}\right)x}$$

### 2.4. Life Table Construction

Life tables were constructed using the methods described by Jandricic et al. [20], Birch [35] and Southwood and Henderson [36]. Data for the survival, birth, and development of individuals from the neonate nymph stage to death at eight different temperatures between 5 and 27.5 °C were collected. The age-specific survival rate (*l_x_*) (Equation (9)) and the survival rate (*s_xj_*) were defined to be the probability that a newly born individual would survive to age *x* and stage *j*, respectively. The *s_xj_* provides the beginning age *x* of the survival curve of stage *j*, last age *x* of the survival curve of stage *j*, mortality of stage *j*, and death age of individuals that died in stage *j*. The death, the beginning, and end of a stage can be provided.

(9) $$l_{x}=\sum_{j=1}^{k}S_{xj}$$

where *k* is the number of stages.

The age-specific fecundity (*b_x_*) (Equation (10)) was calculated to take individuals of different stages at age *x* into account. The age–stage-specific fecundity (*f_xj_*) is the number of nymph eggs produced by a female adult at age *x*.

(10) $$b_{x}=\frac{\sum_{j=1}^{k}S_{xj}f_{xj}}{\sum_{j=1}^{k}S_{xj}}$$

Net reproductive rate (*R*_0_) was calculated as $R_{O}=\sum_{x=0}^{\infty }l_{x}b_{x}$, being the total number of offspring that an individual can produce during its lifetime. Traditionally, the intrinsic rate of increase (*r*_m_) was obtained with a Euler equation, ∑[e^(−*r*m × *x*)^ × *lx* × *bx*], and, when summed across all ages, the values in this equation added up almost to 1 (calculated to ten decimal points); this calculation was performed using Microsoft Excel (Microsoft Office Professional Plus 2013). The finite rate of increase (λ), generation time (G) (in days), and doubling time (DT) (in days) were calculated as e*^r^*^m^, Log_e_(*R*_0_)/*r*_m_ and Log_e_(2)/*r*_m_, respectively.

The life history data of *A. solani* were also analyzed based on the age–stage, two-sex life table theory [22] and the method described by Chi [23] using the computer program TWOSEX-MSChart [37]. The intrinsic rate of increase (*r*) is calculated using the Euler–Lotka formula (Equation (11)) with age indexed from Day 0 [38], as follows.

(11) $$\sum_{x=0}^{\infty }e^{-r\left(x+1\right)}l_{x}m_{x}=1$$

The generation time (G) (in days), and doubling time (DT) (in days) were calculated as ln(*R*_0_)/*r*_m_ and ln(2)/*r*_m_, respectively. The statistical differences among different temperatures were analyzed with a paired bootstrap test at the 5% significance level [39]. The bootstrap method was contained in the computer program TWOSEX-MSChart. The life table data were used to predict and compare the population growth of *A. solani* at different temperatures using the TIMING-MSChart program [40]. The 10 newborn nymphs were the starting point used to simulate the population growth of each temperature. The common logarithm was used to describe the population growth of stage *j* from time *t* to *t* + 1.

## 3. Results

### 3.1. Nymphal and Adult Development and Fecundity

*Aulacorthum solani* nymphs could not complete development to adult emergence at 2.5 or at 30 °C. Their mortality at 5 and 27.5 °C, however, was very low, and had values of 10% and 0%, respectively. The mortality at 7.5 °C increased to ca. 23%, but decreased again from 3.3% to 0% with increasing temperature. At all temperatures at which nymphs completed development, only four nymphal instars were observed. Nymphs showed high stage-specific mortalities, ca. 73%, in the first instar stage at 2.5 °C, while at 30 °C, they showed a high mortality of 63% in the second instar. The length of the nymphal period, including the duration of each separate instar, decreased with increasing temperature, but the durations of the first, second, and third instars increased at 30 °C (Table 1).

Adult longevity was longer at lower temperatures over the range of 5.0 to 12.5 °C, and thereafter decreased with increasing temperature; lifespans were shortest (3.7 days) at 30 °C. Reproductive rates were high at all temperatures. The length of the pre-reproductive period decreased with increasing temperature from 5 to 25 °C, but then increased slightly at 27.5 °C, and then decreased again at 30 °C. The reproductive period was the longest at 7.5 and 12.5 °C, and it decreased with increasing temperature within the upper range of the temperatures tested. Total fecundity was significantly higher from 12.5 to 20 °C compared with that at lower and higher temperatures. Mean daily fecundity during the reproductive period was the highest at 20 °C and 25 °C, with 3.8 and 3.6 nymphs produced per adult per day, respectively, at these temperatures (Table 2). Notably, only one adult out of the thirty apterous adults exposed to 30 °C changed to the alate form two days after emergence and before death (longevity: 4 days), without undergoing parthenogenesis.

### 3.2. Estimation of Developmental and Reproductive Patterns Based on Models

**Figure 1:**
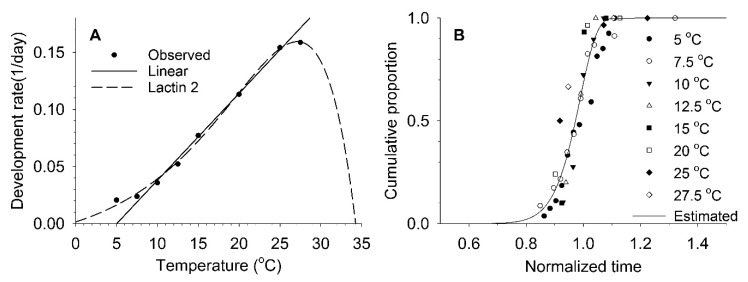
Observed values of development rates at constant temperatures and estimated linear (Equation (1)) and nonlinear Lactin-2 model (Equation (2)) curves between temperature and development rates (**A**), and Weibull distribution curve (Equation (3)) for development completion (**B**) in *A. solani* nymphs.

LDT and the degree-days (DD) above LDT required to complete nymphal development (i.e., the thermal constant) were determined to be 5.02 °C and 131.2 DD, respectively, based on the linear development model (Equation (1)) (Table 3 and Figure 1). The LDTs of each separate nymphal instar estimated using the linear model ranged from 5.39 to 6.32 °C (Table 4). The LDT of the nymphal stage overall determined using the Lactin-2 model (Equation (2)) was −1.85 °C (Table 3). The estimated upper lethal temperature (UDT) was 33.9 C. The optimal temperature was estimated to be 26.87 °C. The two development rate models had very high adjusted coefficients of determination (*r*_adj_^2^) values, which were ≥0.99 for both models. The data for inter-individual variation in developmental completion were fit well by the model tested (Equation (3)), with a high *r*_adj_^2^ value of 0.90 (Table 3 and Figure 1). The model parameters and values derived from this model for each nymphal instar are provided in Table 4.

**Figure 2:**
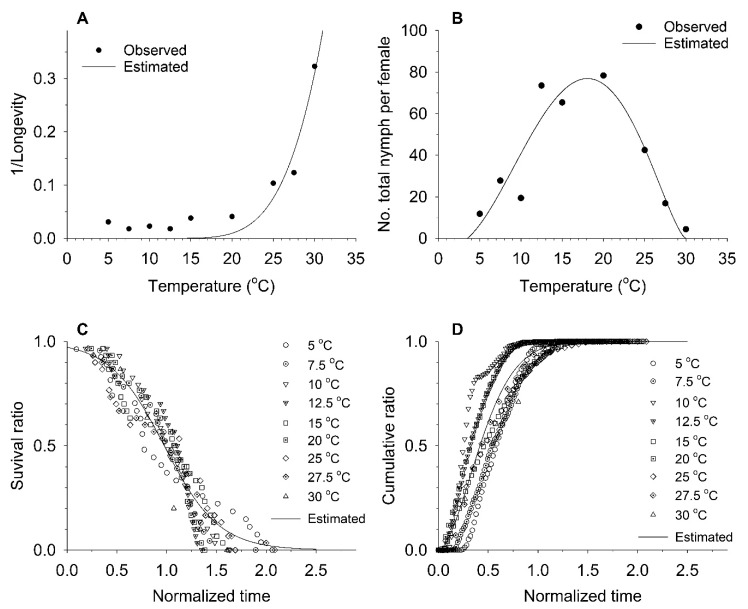
Observed values of the aging rate (**A**), total fecundity (**B**), cumulative survival ratio (**C**), and cumulative fecundity ratio (**D**), and curves estimated from their generalized models (Equation (4), Equation (5), Equation (6), Equation (3)) in *A. solani* adults.

Aging rate increased exponentially with increasing temperature, and its generalized model (Equation (4)) achieved a high *r*_adj_^2^ value of 0.88 (Table 5 and Figure 2). However, the model underestimated observed aging rates between 5 and 20 °C. The total fecundity model (Equation (5)) achieved an *r*_adj_^2^ value of 0.74, and estimated that the maximum fecundity of this species on soybean is ca. 76.9 nymphs per adult at an optimum temperature of 18.1 °C. Survival (Equation (6)) and cumulative fecundity (Equation (3)) models applied to the normalized ages of adults fit the observed values well, with high *r*_adj_^2^ values.

**Figure 3:**
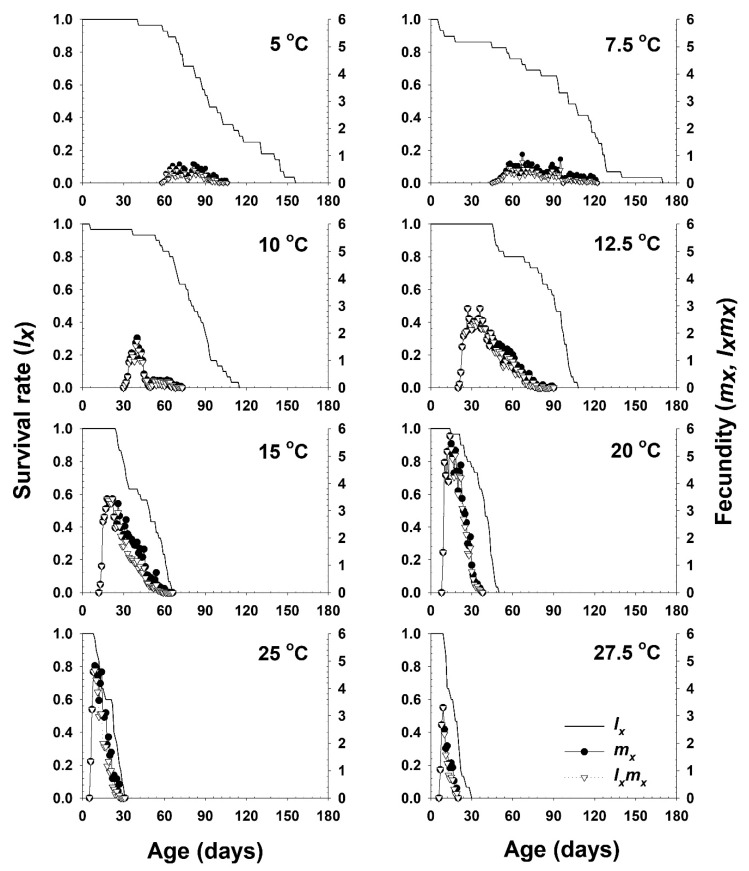
The age-specific survival rate (*l_x_*), the age-specific fecundity (*m_x_*), and the age-specific maternity (*l_x_m_x_*) of *A. solani* in response to different temperature conditions.

The age-dependent daily mortality of adults at particular temperatures rapidly decreased with increasing temperature. The age-specific survival rate and fecundity of *A. solani* are presented in Figure 3. The age-specific survival rate (*l_x_*) is the sum of *s_xj_* at each age *x* and is the simplified version of *s_xj_*. The curves sharply dropped at an earlier age from 15 °C to 27.5 °C. The daily fecundity at temperatures from 10 to 27.5 °C sharply increased at earlier ages, and slowly decreased thereafter until the final parthenogenesis occurred, while at lower temperatures of 5 and 7.5 °C, daily fecundity values were relatively stable across ages. The highest age-specific fecundities were 0.70 (81 d), 1.04 (67 d), 1.82 (40 d), 2.90 (36 d), 3.43 (22 d), 5.73 (14 d), 4.82 (9 d), and 3.30 (9 d) offspring at 5, 7.5, 10, 12.5, 15, 20, 25, and 27.5 °C, respectively. The peaks of *m_x_* and *l_x_m_x_* tended to increase with increasing temperature from 5 °C to 20 °C. The model (Equation (7)) used to generalize the relationship between adult age and daily fecundity fit the observed data well at temperatures between 12.5 and 25 °C, with a high *r*_adj_^2^ value over 0.9. The performance of this model at lower (5~10 °C) and higher temperatures (27.5 and 30 °C) than those in this range, however, was relatively poor (Table 6). The model estimated the highest fecundity to be ca. 88.5 nymphs per adult at 20 °C. However, the estimated pre-reproductive periods (*p*_2_) in the model were shorter than the observed periods at all temperatures. Although the *p*_2_ parameter of the model at 25 °C gave a negative value, it was close to zero. The maximum daily fecundity was estimated to be ca. 6.1 nymphs per adult for a 5.2 day old of adult at 21.3 °C in the age- and temperature-dependent daily fecundity model (Equation (8)) (Table 7).

### 3.3. Life Table

The intrinsic rate of increase (*r*_m_) and the finite rate of increase (λ) from both analyses were highest at 25 °C (0.3349, 0.3377, and 1.40, respectively). Doubling time (DT) was shortest at 25 °C (2.07 and 2.05 days). Net reproductive rate (*R*_0_) was highest at 20 °C (77.49 and 78.34 nymphs), and the temperature with the second-highest *R*_0_ value was 12.5 °C (73.19 and 73.56 nymphs) (Table 8 and Table 9). The generation time (G) decreased with increasing temperature.

**Figure 4:**
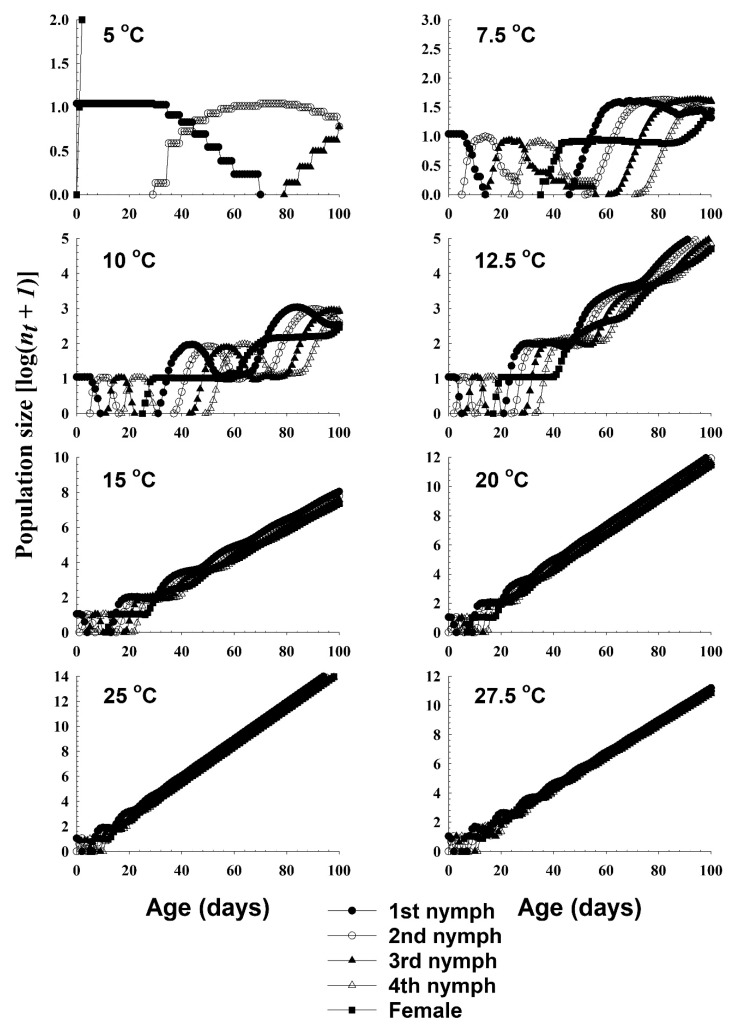
The population growth projection of *A. solani* in response to different temperature conditions, beginning with an initial population of newly born 10 nymphs.

The population growth and stage structure at different temperatures based on the age–stage, two-sex life table is presented in Figure 4. The populations from 20 °C and 25 °C increased significantly faster than those at lower temperature conditions.

## 4. Discussion

The development of insects can be affected by the nutritional value of their host plant, as well as by temperature. The duration of the nymphal period of *A. solani* on different host plants, including eggplant (*Solanum melongena* L. (Solanales: Solanaceae)), fennel (*Foeniculum vulgare* Mill (Apiales: Apiaceae)), lettuce, pea (*Pisum sativum* L. (Fabales: Fabaceae)), pansy, potato (*Solanum tuberosum* L. (Solanales: Solanaceae)), and soybean, are as follows: 48.7–63.15 days at 5 °C, 16.7–28.0 days at 10 °C, 16.9–19.2 days at 12.5 °C, 10.3–13.4 days at 15 °C, 7.2–8.9 days at 20 °C, 6.5–7.4 days at 25 °C, 6.3 days at 27.5 °C (data from this study and Jandricic et al. [20]). The nymphal period of *A. solani* on the same plant species, soybean, as that used in our study was previously observed to be ca. 20 days at 10 °C by Kim et al. [2], which differed considerably from our result (28 days) at this temperature; however, the results of our study and theirs at other common temperatures were not so different (Table 1). The difference of development time at 10 °C may have been due to the use of different cultivars (Taegwangkong and Paldalkong) in the two studies.

The lowest experimental temperature tested in this study was 2.5 °C, which caused 100% mortality of *A. solani* nymphs on soybean (Table 1). Similarly, Pozarowska [41] reported no survival of nymphs of this species kept at 2 °C on potato. LDTs estimated for this species with the linear model were 5.02 °C on soybean in this study (Table 3), 4.8 °C on lettuce [19], and 3.69 °C on pansy [20]. LDTs estimated using the linear model are usually different depending on the temperature range used, even within the same experiment, and thus the thermal constants for the completion of development estimated also vary along with these different LDTs. Other estimated LDTs on lettuce were 1.09 °C and 0.08 °C [16,17]. Generally, the LDTs for aphid species are lower than those for members of other insect orders. Indeed, based on data published in the literature for 28 cases from 16 species of aphids, the mean ± SD values of LDT and DD for the completion of development estimated from the linear model were 4.1 ± 1.6 °C and 131.4 ± 38.3 DD, respectively [10]. These values cover all of the LDTs and DDs estimated for the nymphal stages of *A. solani* in this study (Table 1 and Table 4). The Lactin-2 model estimated the LDT to be −1.85 °C in this study (Table 3), but this value is probably not realistic because we observed that all nymphs died at the much higher temperature of 2.5 °C. The LDT of this species on pansy was previously estimated with the Lactin-2 model to be 4.0 °C [20], which is more realistic. The low LDTs estimated for *A. solani* indicate that this aphid is adapted to lower temperatures, and as a result of this, its survival rates during the winter in temperate regions should be relatively high. Indeed, the survival of anholocyclic forms of *A. solani* on weeds over the winter season in Scotland was previously reported [9].

Generally, the development of insects is retarded at temperatures above their optimal temperature. In this study, the nymphal period was not retarded by increasing temperature between 5 and 27.5 °C, and survival was high at all these temperatures. However, the developmental periods of the first, second, and third nymphal instars at 30 °C were longer than those at 27.5 °C. The duration of the fourth instar at 27.5 °C was also longer than that at 25 °C, although this difference was not statistically significant (Table 1). This property was also observed in other studies of *A. solani*, in which longer nymphal periods occurred at 25 °C than 22.5 °C on lettuce [17], at 28 °C than 25 °C on lettuce [16], and at 30 °C than 25 °C on pansy [20].

At the highest temperature tested in this study, 30 °C, mortality during the nymphal period was 100%, which agreed with the results of two previous studies also conducted on soybean [2,42]. Data for nymphs raised on other host plants are also similar, as 100% mortality was reported to occur at 31 °C on lettuce [16] and high mortality, ca. 88%, at 30 °C and 100% mortality at 35 °C was reported on pansy [20]. However, a study on lettuce produced a different result, in that ca. 97% mortality occurred at 27.5 °C [17]. The Lactin-2 model estimated the value of this species’ upper developmental threshold to be 33.9 °C, which was higher than the range of temperatures observed in this study (Table 1 and Table 3). For nymphs reared on pansy, this parameter was estimated to be 37.6 °C based on the Lactin-2 model, and this value was also higher than the observed temperatures at which high or total mortality occurred [20].

The optimal temperature estimated using the Lactin-2 model for the nymphal development of *A. solani* on soybean was 26.87 °C in this study, while the optimal temperature for nymphs reared on pansy was previously estimated to be 25.7 °C based on the Lactin-2 model [20].

The range of temperatures (12.5–20 °C) over which we found higher fecundities was similar to that found in other studies, which was 16–22 °C and 12.5–20 °C on lettuce, and 10–20 °C on pansy [15,17,20]. The maximum total fecundities of *A. solani* estimated were commonly below 90 nymphs per adult in all previous studies, which agrees with our results. However, it appears that high temperatures above 20 °C can suppress the reproduction of *A. solani*, since we observed that the total fecundity and net reproductive rate (*R*_0_) decreased significantly at 25 °C compared to at 20 °C, at which the highest fecundity and *R*_0_ values were found (Table 2 and Table 6). Similar results were found in other studies, wherein total fecundity and *R*_0_ on lettuce were ca. 68 and 63 nymphs per adult, respectively, at 22 °C, but ca. 18 and 23 nymphs at 25 °C [15]; on pansy, these were ca. 68 and ca. 64 nymphs at 20 °C, but ca. 39 and ca. 37 nymphs at 25 °C [20]. However, these findings should be further confirmed, because Kim et al. [2]’s study rearing this species on soybean showed that the highest fecundity, ca. 83 nymphs per adult, occurred at 25 °C.

Several characteristics of the adult biology of *A. solani* on soybean in the study described in Kim et al. [2] were quite different from the results of this study. Adult longevities and reproductive periods at 25 °C and 30 °C were much longer in Kim et al. [2] than those in this study. Total fecundities at 10 and 30 °C were also much higher in the previous study [2]. Fecundity is an ideal biological trait for demonstrating the quality of a particular host by measuring the reproductive success of the species. It may be inferred that the differences were caused by the use of different soybean cultivars, and aphid incubation methods, experimental conditions, and differences in the local populations of the aphid in the two studies. Montllor et al. [43] found that when *Acythosiphon pisum* had been heat-stressed for a period of about 4 h at 39 °C as young (1 day) or as older (5 day) nymphs, *A. pisum* without pea aphid secondary symbiont (PASS) or pea aphid rickettsia (PAR) failed to reproduce, though not necessarily to survive. Although we did not identify the facultative bacterial secondary symbionts of *A. solani* in this study, it may be speculated there is a relationship between development and the facultative symbionts of *A. solani*, because the species could not develop the adult stage at 30 °C and 35 °C but they reproduced at the same temperature.

Life table parameters (the intrinsic rate of increase, finite rate of increase, and net reproductive rate) are appropriate indicators for evaluating the effect of host plants on the development, survival, and fecundity of different populations. The intrinsic rate of increase of this study was higher than that of De Conti et al. [15], but lower than that of Jandricic et al. [20]. The values under lower temperatures (12.5 and 15 °C) in this study were lower and the values at 20 and 25 °C were higher than those of Lee et al. [18]. The aphids may be impaired reproduction and produce few nymphs when fed on different host plant leaves containing low or high levels of soluble nitrogen [44,45,46]. This study presented the advantages of using the age–stage, two-sex life table theory in describing demography. We used survival rate and fecundity data to project the population growth of *A. solani* to show the advantage of using the age–stage, two-sex life table in revealing the stage structure of insect populations.

## 5. Conclusions

We investigated several characteristics of the development and reproduction of *A. solani* on soybean at 10 different temperatures, and described the relationship between temperature and this species’ biology. Our results indicated that *A. solani* can survive and reproduce on soybean over a temperature range from 5 to 27.5 °C. High rates of reproduction occurred from 12.5 to 20 °C. The estimated LDT, UDT, and *T*_opt_ for the nymphal development of this species were ca. 5.02 °C, 33.7 °C, and 26.9 °C, respectively. The estimated total fecundity was ca. 76.9 nymphs per adult at 18.1 °C. Most models and their parameters used in this study fitted the observed data well. These models and the life table statistics derived herein should, however, be evaluated under conditions of fluctuating temperatures in the field or in field-simulating conditions before they are applied in future population dynamics studies.

## Figures and Tables

**Table 1:** Development duration (days, D) (mean ± SD) and stage-specific mortality (%, M) of *Aulacorthum solani* nymphs on soybean at constant temperatures.

Temp (°C)	First Instar		Second Instar		Third Instar		Fourth Instar		Total Nymph			
	This Study		This Study		This Study		This Study		This Study		Kim et al. [2] ^2^	
	D	M	D	M	D	M	D	M	D	M	D^2^	M^2^
2.5	16.9 ± 6.2 a ^1^	73.3	25.0 ± 16.5 a ^1^	62.5	-	100.0	-	-	-	100.0		
5.0	9.3 ± 2.3 b	3.3	12.0 ± 1.3 b	3.4	13.4 ± 1.7 a ^1^	0.0	14.3 ± 2.0 a ^1^	3.6	48.7 ± 3.4 a ^1^	10.0		
7.5	8.9 ± 2.2 b	3.3	10.7 ± 1.8 b	10.3	12.0 ± 5.5 a	3.8	12.3 ± 1.6 b	8.0	42.4 ± 5.0 b	23.3		
10.0	7.2 ± 1.1 c	0.0	6.7 ± 0.6 c	3.3	6.2 ± 0.4 b	0.0	7.9 ± 0.5 c	0.0	28.0 ± 1.1 c	3.3	20.2 ± 4.5	3.0
12.5	4.1 ± 0.5 d	0.0	4.6 ± 0.5 cd	0.0	4.6 ± 0.6 bc	0.0	5.8 ± 0.6 d	0.0	19.2 ± 0.7 d	0.0		
15.0	3.2 ± 0.6 de	0.0	3.0 ± 0.3 de	0.0	3.1 ± 0.4 bcd	0.0	3.7 ± 0.5 e	0.0	13.0 ± 0.4 e	0.0	13.4 ± 2.6	3.0
20.0	2.2 ± 0.5 ef	0.0	2.1 ± 0.3 e	0.0	2.0 ± 0.3 cd	0.0	2.6 ± 0.5 f	0.0	8.9 ± 0.6 f	0.0	7.8 ± 1.2	0.0
25.0	1.7 ± 0.5 f	0.0	1.7 ± 0.5 e	0.0	1.4 ± 0.5 d	0.0	1.8 ± 0.4 g	0.0	6.5 ± 0.6 g	0.0	7.0 ± 1.0	0.0
27.5	1.6 ± 0.5 f	0.0	1.4 ± 0.5 e	0.0	1.3 ± 0.5 d	0.0	2.0 ± 0.3 fg	0.0	6.3 ± 0.5 g	0.0		
30.0	2.0 ± 0.9 ef	0.0	2.1 ± 0.7 e	63.3	5.0 bc	90.9	-	100.0	-	100.0	-	100

^1^ Thirty neonate nymphs were treated at various temperatures. Different letters within a column indicate statistically significant difference among means by Tukey’s HSD test at *α* = 0.05. ANOVA results were *F*_9,266_ = 161.47 (*p* < 0.0001) for 1st instar nymph, *F*_9,237_ = 173.47 (*p* < 0.0001) for 2nd, *F*_8,224_ = 145.79 (*p* < 0.0001) for 3rd, *F*_7,221_ = 707.03 (*p* < 0.0001) for 4th, and *F*_7,221_ = 1,741.79 (*p* < 0.0001) for total nymphal period. ^2^ These data are from soybean in Kim et al. [2], where 20 nymphs were replicated and Paldalkong cultivar was used.

**Table 2:** Developmental duration (days) and fecundity of *A. solani* adults on soybean at constant temperatures.

Temp (°C)	Init. No. ^1^	Longevity (Day)		Rep. Rate (%) ^3^	R. No.	Pre-Reproductive Period (Day)	Reproductive Period (Day)		Total Fecundity (No.)		Daily Fecundity
		This Study	Kim et al. [2] ^4^	This Study		This Study	This Study	Kim et al. [2] ^4^	This Study	Kim et al. [2] ^4^	This Study
5.0	27	52.9 ± 30.1 b^5^		92.6	25	17.4 ± 2.7 a	22.4 ± 12.1 b		11.8 ± 5.8 ef		0.6 ± 0.2 d
7.5	23	67.0 ± 25.3 a		82.6	19	12.8 ± 1.4 b	45.9 ± 19.4 a		27.8 ± 12.4 d		0.6 ± 0.2 d
10.0	29	53.2 ± 17.8 b	52.6 ± 26.7	100.0	29	7.1 ± 0.8 c	24.5 ± 9.6 b	34.8 ± 10.4	19.4 ± 5.2 de	41.8 ± 23.6	0.9 ± 0.3 d
12.5	30	65.1 ± 20.4 ab		100.0	30	4.6 ± 0.6 d	46.0 ± 12.6 a		73.5 ± 12.9 ab		1.7 ± 0.3 c
15.0	30	33.3 ± 14.3 c	42.4 ± 21.4	100.0	30	2.6 ± 0.5 e	28.4 ± 11.9 b	28.7 ± 8.3	65.4 ± 20.9 b	58.9 ± 25.7	2.4 ± 0.4 b
20.0	30	28.9 ± 9.0 c	31.4 ± 12.5	100.0	30	1.8 ± 0.4 ef	21.7 ± 5.9 b	26.4 ± 5.2	78.3 ± 14.6 a	78.9 ± 19.4	3.8 ± 0.6 a
25.0	30	14.2 ± 7.1 d	28.2 ± 4.3	100.0	30	1.3 ± 0.5 f	12.4 ± 6.3 c	23.8 ± 3.6	42.5 ± 18.8 c	83.0 ± 21.5	3.6 ± 0.7 a
27.5	30	11.5 ± 6.0 d		93.3	28	2.5 ± 0.6 e	7.3 ± 3.6 cd		16.9 ± 9.3 de		2.3 ± 0.8 b
30.0 ^2^	30	3.7 ± 1.3 d	13.3 ± 5.7	80.0	24	1.5 ± 0.6 f	2.6 ± 1.2 d	11.2 ± 6.1	4.4 ± 2.3 f	25.4 ± 14.8	1.7 ± 0.5 c
35.0			4.2 ± 2.1					2.3 ± 1.8		2.5 ± 1.7	

^1^ Initial number means the number of adults that emerged from nymphal stage. ^2^ The adults at 30 °C were treated separately without examination of their nymphal stages. ^3^ Reproductive rate was calculated as the percentage of the initial number of adults that produced progeny. ^4^ These data are from soybean in Kim et al. [2], in which 20 adults were replicated and Paldalkong cultivar was used. ^5^ Different letters within a column indicate statistically significant difference among means by Tukey’s HSD test at *α* = 0.05. ANOVA results were *F*_8,250_ = 58.09 (*p* < 0.0001) for longevity, *F*_8,236_ = 677.57 (*p* < 0.0001) for pre-reproductive period, *F*_8,236_ = 57.93 (*p* < 0.0001) for reproductive period, *F*_8,236_ = 128.48 (*p* < 0.0001) for total fecundity, and *F*_8,236_ = 158.57 (*p* < 0.0001) for daily fecundity during reproductive period.

**Table 3:** Parameters and values derived from the development rate models and the distribution model for *A. solani* nymphs in Figure 1.

Model	Parameters and Derived Estimates	*r*_adj_^2^
Developmental rate Linear (Equation (1))	*a* = 0.00762 ± 0.00026, *b* = -0.03824 ± 0.00420 (Temperature range 7.5~25 °C), LDT = 5.02, DD = 131.19	0.99
Lactin-2 (Equation (2))	*Ρ* = 0.14202 ± 0.01970, *T*_L_ (UDT) = 33.88565 ± 1.61807, *ΔT* = 7.01425 ± 0.95905, *λ* = −0.01491 ± 0.01322, LDT = −1.85, *T*_opt_ = 26.87	0.99
Weibull distribution (Equation (3))	*η* = 0.99247 ± 0.00444, *β* = 17.94321 ± 1.87829	0.90

ANOVA results were *F*_1,4_ = 857.0 (*p* < 0.0001) for the linear model, *F*_3,4_ = 448.7 (*p* < 0.0001) for the Lactin-2 model, and *F*_1,40_ = 365.0 (*p* < 0.0001) for the Weibull distribution model. Abbreviations: LDT, lower development threshold; DD, degree-days; UDT, upper development threshold; *r*_adj_^2^, adjusted *r* square value.

**Table 4:** Parameters and values derived from the linear and nonlinear temperature-dependent development models in separate instars of *A. solani* nymphs.

Model	Parameters and Derived Values	First Instar	Second Instar	Third Instar	Fourth Instar
Linear	*a* (slope)	0.03351 ± 0.00093	0.03579 ± 0.00151	0.04072 ± 0.00336	0.0298 ± 0.00178
	*b* (intercept)	−0.18052 ± 0.01608	−0.20268 ± 0.02740	−0.25756 ± 0.05424	−0.1702 ± 0.02871
	Temp. range(°C)	10.0–25.0	7.5–27.5	7.5–25.0	7.5–25.0
	LDT and DD	5.39, 29.8	5.66, 27.9	6.32, 24.6	5.70, 33.5
	*r*_adj._^2^	1.00	0.99	0.96	0.98
	ANOVA	*F*_1,3_ = 1308.8 (*p* < 0.0001)	*F*_1,5_ = 565.0 (*p* < 0.0001)	*F*_1,4_ = 146.8 (*p* = 0.0003)	*F*_1,4_ = 281.1 (*p* < 0.0001)
Lactin-2	*Ρ*	0.14597 ± 0.01197	0.18764 ± 0.02063	0.23443 ± 0.01455	0.19659 ± 0.02063
	*T*_L_ (UDT)	33.78729 ± 0.60461	32.08891 ± 0.51063	30.30548 ± 0.11484	30.34261 ± 0.60381
	*ΔT*	6.75625 ± 0.52066	5.30357 ± 0.56751	4.25799 ± 0.26086	5.06637 ± 0.52048
	*λ*	−0.024430 ± 0.03485	0.02808 ± 0.04283	0.06220 ± 0.03000	0.01504 ± 0.03185
	LDT	−8.36	- ^1^	- ^1^	- ^1^
	*T*_opt_	27.03	26.79	26.05	25.28
	*r*_adj._^2^	0.99	0.97	0.98	0.99
	ANOVA	*F*_3,6_ = 409.1 (*p* < 0.0001)	*F*_3,6_ = 121.3 (*p* < 0.0001)	*F*_3,5_ = 188.2 (*p* < 0.0001)	*F*_3,4_ = 214.2 (*p* < 0.0001)
Weibull	*η*	0.96899 ± 0.01653	0.94956 ± 0.01867	0.91102 ± 0.02093	0.96280 ± 0.00995
distribution	*β*	3.91272 ± 0.38530	7.50896 ± 1.75786	5.59675 ± 1.07504	10.54906 ± 1.46181
	*r*_adj._^2^	0.89	0.73	0.78	0.90
	ANOVA	*F*_1,43_ = 363.5 (*p* < 0.0001)	*F*_1,30_ = 90.3 (*p* < 0.0001)	*F*_1,28_ = 111.4 (*p* < 0.0001)	*F*_1,27_ = 273.5 (*p* < 0.0001)

^1^ The parameters could not be calculated. Abbreviation: *T*_opt_, optimal temperature.

**Table 5:** Several models and their parameters for *A. solani* adults in Figure 3.

Model	Parameters	*r*_adj_^2^
Aging rate (Equation (4))	*a* = 4.70782 ± 7.95564, *b* = 183.66418 ± 49.24300	0.88
Total fecundity (Equation (5))	*a* = 0.0074 ± 0.0182, *T_L_* = 3.4950 ± 3.0050, *T_M_* = 30.0000 ± 3.7360, *m* = 0.6756 ± 0.3867	0.74
Survival ratio (Equation (6))	*γ* = 0.97594 ± 0.00988, *δ* = −0.27363 ± 0.01034	0.92
Cumulative fecundity ratio (Equation (3))	*η* = 0.51445 ± 0.00655, *β* = 1.73794 ± 0.05582	0.91

ANOVA results were *F*_1,7_ = 70.96 (*p* < 0.0001) for aging rate, *F*_3,5_ = 11.17 (*p* = 0.0177) for total fecundity, *F*_1,174_ = 1949.19 (*p* < 0.0001) for survival ratio, and *F*_1,566_ = 5751.26 (*p* < 0.0001) for cumulative fecundity ratio.

**Table 6:** Parameters estimated from the daily fecundity model (Equation (7)) of *A. solani* adults at constant temperatures.

Temp (°C)	*p*_1_	*p*_2_	*p*_3_	*r*_adj._^2^
5.0	18.12654 ± 1.31138	3.98639 ± 0.68477	0.05573 ± 0.00382	0.58
7.5	31.87245 ± 1.52136	3.74750 ± 0.60615	0.04654 ± 0.00221	0.70
10.0	22.88171 ± 1.72476	1.52812 ± 0.33140	0.12175 ± 0.00871	0.66
12.5	83.24260 ± 1.99011	1.21637 ± 0.20426	0.08076 ± 0.00201	0.93
15.0	83.47867 ± 2.64907	0.22761 ± 0.24133	0.10680 ± 0.00374	0.91
20.0	88.53896 ± 3.64552	0.18145 ± 0.17975	0.17008 ± 0.00750	0.91
25.0	57.06038 ± 2.09382	−0.04473 ± 0.12002	0.23537 ± 0.00946	0.94
27.5	22.04102 ± 1.87325	0.30660 ± 0.15650	0.30116 ± 0.02514	0.81
30.0	4.90767 ± 0.85356	0.00785 ± 0.13275	0.64841 ± 0.12095	0.61

ANOVA results were *F*_2,107_ = 76.82 (*p* < 0.0001) for 5 °C, *F*_2,125_ = 148.62 (*p* < 0.0001) for 7.5 °C, *F*_2,85_ = 88.41 (*p* < 0.0001) for 10 °C, *F*_2,86_ = 627.95 (*p* < 0.0001) for 12.5 °C, *F*_2,52_ = 263.86 (*p* < 0.0001) for 15 °C, *F*_2,38_ = 208.32 (*p* < 0.0001) for 20 °C, *F*_2,22_ = 197.73 (*p* < 0.0001) for 25 °C, *F*_2,22_ = 56.01 (*p* < 0.0001) for 27.5 °C, and *F*_2,4_ = 8.32 (*p* = 0.0375) for 30 °C.

**Table 7:** Parameters estimated from the age- and temperature-dependent daily fecundity model (Equation (8)) of *A. solani* adults.

Parameter Estimates	*r*_adj._^2^
*ε* = −3.42273 ± 0.11850, *θ* = 0.02574 ± 0.00096, *κ* = −0.00074 ± 0.00003, *ρ* = −3.15503 ± 0.06940, *ν* = 0.00574 ± 0.00057, *ψ* = −0.00012 ± 0.00002	0.90

ANOVA result is *F*_5,562_ = 981.4 (*p* < 0.0001).

**Table 8:** Life table parameters of *A. solani* at different constant temperatures using traditional age-specific life table analysis.

Temp. (°C)	*r*_m_	*R*_0_ (No.)	G (Day)	λ	DT (Day)
5.0	0.0297	9.61	76.29	1.03	23.37
7.5	0.0405	18.51	72.08	1.04	17.12
10.0	0.0699	18.71	41.91	1.07	9.92
12.5	0.1226	73.19	35.02	1.13	5.65
15.0	0.1813	64.21	22.95	1.20	3.82
20.0	0.2774	77.49	15.68	1.32	2.50
25.0	0.3349	40.67	11.06	1.40	2.07
27.5	0.2489	14.79	10.82	1.28	2.79

*r*_m_: intrinsic rate of increase, *R*_0_: net reproductive rate in numbers, G: generation time in days, λ: finite rate of increase, and DT: doubling time of population in days.

**Table 9:** Life table parameters of *A. solani* at different constant temperatures using age–stage, two-sex life table analysis.

Temp. (°C)	*r*_m_	*R*_0_ (No.)	G (Day)	λ	DT (Day)
5.0	0.0307 h	10.51 e	76.36 a	1.03 h	22.61 a
7.5	0.0402 g	18.17 d	71.85 b	1.04 g	17.32 b
10.0	0.0689 f	17.68 d	41.66 c	1.07 f	10.06 c
12.5	0.1226 e	73.56 ab	35.05 d	1.13 e	5.65 d
15.0	0.1827 d	65.29 b	22.86 e	1.20 d	3.79 e
20.0	0.2774 b	78.34 a	15.72 f	1.32 b	2.50 g
25.0	0.3377 a	42.52 c	11.09 g	1.40 a	2.05 h
27.5	0.2534 c	15.79 d	10.87 g	1.28 c	2.74 f

*r*_m_: intrinsic rate of increase, *R*_0_: net reproductive rate in numbers, G: generation time in days, λ: finite rate of increase, and DT: doubling time of population in days. Means in the same row followed by different letters are significantly different (*p* < 0.05), as determined by the paired bootstrap test.
